# Does Gender Influence Physiological Tolerance in Resuscitators When Using Personal Protection Equipment against Biological Hazards?

**DOI:** 10.1155/2018/5890535

**Published:** 2018-10-16

**Authors:** Francisco Martín-Rodríguez, José Luis Martín Conty, Verónica Casado Vicente, Pedro Arnillas Gómez, Alicia Mohedano-Moriano, Miguel Ángel Castro Villamor

**Affiliations:** ^1^Advanced Clinical Simulation Center, Department of Medicine, Dermatology and Toxicology, Faculty of Medicine, Valladolid, Emergency Medical Services, SACYL, Castilla y León, Avda. Ramón y Cajal, 7, 47005 Valladolid, Spain; ^2^Faculty of Occupational Therapy, Speech Therapy and Nursing, University of Castilla la Mancha, Avda. Real Fábrica de Seda, s/n 45600, Talavera de la Reina, Toledo, Spain; ^3^Teaching Unit of Family and Community Medicine, Department of Medicine, Dermatology and Toxicology, Faculty of Medicine, Avda. Ramón y Cajal, 7, 47005 Valladolid, Spain; ^4^Emergency Medical Services, SACYL, Castilla y León, C/Antiguo Hospital Militar s/n, 2^a^ Planta, 47007 Valladolid, Spain

## Abstract

**Introduction:**

Certain professions, such as those related to emergency services, have usually been performed by men, progressively incorporating women into these professions. The main objective of our study was to determine, according to gender, how the use of level D biohazard personal protection equipment (PPE) affects emergency professionals during the performance of resuscitation.

**Materials and Methods:**

An uncontrolled quasi-experimental study was performed on 96 volunteers selected by means of random sampling stratified by gender. Baseline and final vital signs of the assessment activity were analyzed. This activity involves volunteers performing a simulated resuscitation in a controlled environment whilst wearing personal protective suits in a biohazard situation.

**Results:**

Analyzing the physiological tolerance pattern parameter by parameter, and according to* gender*, through a univariate model, we can observe that there is no interaction between tolerance and* gender*; that is, having good or bad tolerance does not depend on* gender. Conclusion*. This specialized skilled work can be performed by any properly trained professional.

## 1. Introduction

For a long time, certain jobs have mainly been held by men. Here we specially refer to prehospital medical emergency services, rescue services, fire extinguishing services, police forces, army personnel, etc. [1]. Women have progressively entered the work market in these specialized professions, in fields where historically most of the workers were men [2, 3]. Currently, in the Degree in Health Sciences, it can be observed that the proportion of male versus female has been reversed, and nowadays more women than men finish a degree in Medicine [4, 5].

Situations such as the recent Ebola virus disease epidemic in West Africa have requested the intervention of many professionals requiring precise technical knowledge, the use of appropriate personal protective equipment against certain risks, and an extra dose of physical effort. However, this equipment is not harmless as it creates mobility, hearing, and vision problems, impairment of thermoregulation, increase in energy demand, etc., thus increasing physiological stress [6, 7].

Emergency services must contemplate within their competency-based curricular design, handling incidents involving biological hazards, including acts of God or resulting from terrorist acts [8–10]. In general terms, we can state that the use of biohazard PPE is especially hard and arduous for workers, imposing a burden of extra physiological stress during the intervention. Hence, we must raise a question: is any worker from the emergency medical services physically prepared to respond to these unique emergency situations? And most importantly, does the worker's gender influence this response? What is more, is there any physiological difference between male and female when responding to an emergency in a biohazard environment whilst wearing protective equipment? It is easy to demonstrate that having to work wearing biohazard PPE makes technical procedures even more difficult [11, 12]. However, there are no extensive studies about the fact that using this equipment requires high-intensity physical effort and high intensity performance that does not allow working long periods of time [13]. It is even more difficult to find studies linking gender with worker's physiological performance during resuscitation.

Performing a conventional cardiopulmonary resuscitation requires skills, technical knowledge, and good physical condition (training) [14]. Performing cardiopulmonary resuscitation in biological risk environments with appropriate protective equipment involves, in addition, extra energy expenditure [15].

The main objective of our study was to determine, according to gender, how the use of level D biohazard personal protection equipment (PPE) affects emergency professionals during the performance of cardiopulmonary resuscitation.

## 2. Materials and Methods

### 2.1. Ethics Statement

The study was approved on 6 April 2016, by the Clinical Research Ethics Committee at Río Hortega University Hospital in Valladolid (Spain), with registration code #412016. All the participants had to read and sign the informed consent, and the entire study was carried out with the highest safety standards, protecting the physical integrity and confidentiality of the participants, complying with national and international regulations for the study in human subjects included in the Helsinki Treaty.

### 2.2. Participants

An uncontrolled quasi-experimental study was carried out on 96 volunteers selected by means of random sampling stratified by* gender* and* academic level* (undergraduate students or Health Sciences professionals) from an opportunity sample of 194 volunteers.

The sample was selected among physicians and nurses of the emergency services and students aged between 18 and 65 years of the Faculty of Health Sciences and who voluntarily decided to participate. Any volunteer presenting at least one exclusion criterion was rejected (Table 1).

### 2.3. Environmental Conditions and Personal Protection Equipment

The simulated resuscitation was performed in a 20 m^2^ laboratory with controlled environmental conditions (average temperature of 33.66°C and average humidity of 51.16 percent).

All the elements of the PPE used in the case study have a CE declaration of conformity and instruction manual. Elements are listed in the sequence for donning PPE according to the recommendations of the European Centre for Disease Prevention and Control (ECDC) [16]. Two pairs of boot covers, a coverall, two pairs of nitrile gloves, hood, FFP3 respirator, and panoramic vented goggles were used.

All the participants in the study had the same prior information and the same equipment and electromedical devices for solving the same case scenario.

### 2.4. Experimental Protocol

#### 2.4.1. Measurement of Baseline Vital Signs

Each participant was asked to sit on a chair, roll up one sleeve, and relax for five minutes, so that the measurements were made at rest. Subsequently, they underwent an electrocardiogram and spirometry to verify normal results. A second stage consisted of serial measurement of vital signs, including* heart rate*,* systolic blood pressure*,* diastolic blood pressure*, t*ympanic temperature*,* total hemoglobin*,* perfusion index*, and baseline* lactic acid levels*.

*Blood pressure* and* heart rate* were measured with the use of oscillometric sphygmomanometry (Schiller BP 200 Plus; Schiller, Baar, Switzerland). For measuring* tympanic temperature* we used a Braun Thermoscan Pro 6000 (Welch Allyn, Inc., New York, USA) with ExacTemp™ technology.* Total hemoglobin* values and* perfusion index* were monitored with a multiparameter monitor Pronto 7 (Masimo, California, USA), software version b99e80000004ef796 (2.2.15), and sensor revised version a83f90f0000c53f2. For the determination of* lactic acid levels* we used an Accutrend Plus meter (Roche Diagnostics, Mannheim, Germany), with a measurement range of 0.8-21.7 mmol/L.

#### 2.4.2. Donning Personal Protective Equipment

Participants put on their PPE following the instructions of a supervisor, according to the donning and removing steps of the European Center for Disease Prevention and Control [16]. The placement and extraction techniques of the PPE and the maneuvers developed during the simulation with the PPE in a specific way were not evaluated, but the physiological response caused by the PPE to the volunteers was evaluated, but it was insisted that the staging and withdrawal techniques should be done according to the protocol and in the most correct way possible.

Participants had ten minutes to fully equip themselves, and they were guided by a specialist throughout the whole process. Then, the equipment was verified to ensure it had been put on correctly.

Each participant had to look at himself or herself in a mirror and check that he or she was properly equipped. Afterwards, they did two squats to check the ergometry and the fit of the equipment, and then they were ready to start the study case.

#### 2.4.3. Advanced Clinical Simulation

A case of cardiac arrest was designed so that each group of four volunteers had to perform resuscitation maneuvers on a simulation mannequin for a period of 20 minutes without stopping, so that they could carry out the appropriate life support techniques, including airway management, defibrillation, vascular access techniques, and chest compressions.

Each group of four volunteers completed the same study case with the same sequence of events. They were reminded that the quality of the procedures would not be assessed, but it was emphasized that all the techniques and procedures had to be performed on the simulation mannequin in the most correct way.

#### 2.4.4. PPE Removal and Subsequent Vital Signs Measurement

Once the participants had finished the case study, a supervised removal of the individual protection equipment was performed and after a 10-minute rest, a new serial measurement of the vital signs took place.

### 2.5. Statistical Analysis

Data was collected and analyzed using Microsoft EXCEL version 14.4.0 and Statistical Product and Service Solutions (SPSS) version 20. Qualitative variables are expressed with their frequency distributions and the quantitative variables as mean and standard deviation (SD). Comparison of means was performed using Student's t-test. Statistical significance was determined where the null-hypothesis (no difference in categories) was rejected with a* p *< 0.05.

## 3. Results

### 3.1. Baseline Values

**Figure 1:**
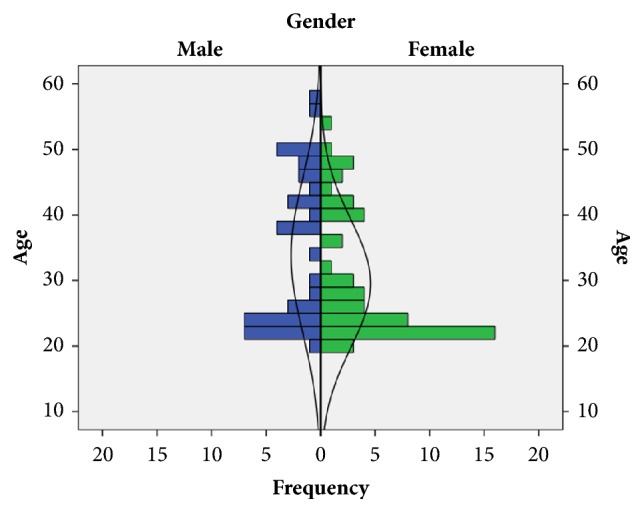
Population pyramid distributed by* gender.*

Of all the subjects who did not meet exclusion criteria, 40 were male (41.7 percent) and 56 female (58.3 percent). Regarding the educational level, 49 subjects (51 percent) were students and 47 (49 percent) professionals of Health Sciences (nurses and physicians). The mean* age* with standard deviation (SD) of participants was 31.31 (10.81) years.  Figure 1 shows* age* distribution by gender. The body mass index (mean ± SD) was of 24.35 (4.12) kg/m^2^. It has been established as poor physiological tolerance to finish the simulated resuscitation having altered 5 or more of the 7 measured parameters. Of the total sample, 74 participants (77.08 percent) showed an acceptable physiological response (4 or less altered parameters), and 22 participants (22.92 percent) had an inadequate response to physiological stress generated during the test (5 or more altered parameters).  Table 2 shows the* age* and body mass index characteristics distributed by gender and physiological tolerance.

### 3.2. Pattern of Poor Physiological Tolerance

Baseline and subsequent measurements were compared in order to create a pattern of poor physiological tolerance after performing the simulated resuscitation whilst wearing biohazard PPE. It is considered a good physiological response finishing the test with lower final values of* heart rate*, systolic or diastolic* blood pressure*,* lactic acid*, and* tympanic temperature* and higher final values of* perfusion index* and* hemoglobin* than baseline ones.

The increase in* heart rate* in final measurements is especially significant, with an average increase of 11.42 bpm (95 percent CI 9.17 to 13.67, *p* < 0.001), as well as in* tympanic temperature*, with an average increase of 0.55°C (95 percent CI 0.46 to 0.64, *p* < 0.001) with respect to the baseline measurements. In both* systolic* and* diastolic blood pressure* values, an average decrease of -4.36 mmHg (95 percent CI -6.96 to -1.77, *p* = 0.001) and -1.59 mmHg (95 percent CI -3.21 to 0.02, *p* = 0.540) was observed with respect to the basal values. Regarding all the rest of the vital signs studied, an increase in* hemoglobin* levels can be observed, with a mean increment of 0.48 mg/dL (95 percent CI 0.26 to 0.69, *p* < 0.001), as well as an increase in the* perfusion index* of 3.28 percent (95 percent CI 2.52 to 4.04, *p* < 0.001) and in* lactic acid* levels of 1.24 mmol/L (95 percent CI 0.74 to 1.75, *p* < 0.001) with respect to the initial measurements. It was established as a cutoff point of poor physiological tolerance, having altered 5 or more of the seven parameters analyzed. The parameters analyzed are* tympanic temperature*,* heart rate*,* systolic blood pressure*,* diastolic blood pressure*,* hemoglobin*,* perfusion index*, and* lactic acid*. Poor toleration was defined by finishing the test with more* tympanic temperature*,* heart rate*,* systolic blood pressure*,* diastolic blood pressure*, and* lactic acid* and with less* hemoglobin* and* perfusion index*, final compared to initial.  Table 3 shows the distribution of vital signs before/after the simulated resuscitation test, based on the built physiological tolerance pattern.

### 3.3. Poor Physiological Tolerance Pattern by Gender

Based on the results mentioned above, a different distribution among the volunteers can be observed depending on the good or bad tolerance to stress caused by the simulated resuscitation. The distribution of vital signs before/after the simulation test according to* gender* shows significant increases in both genders, with mean increases in* heart rate*,* tympanic temperature*, lactic acid,* hemoglobin*, and* perfusion index* and decreases in* systolic *and* diastolic blood pressure* (see Table 4).

Analyzing the physiological tolerance pattern parameter by parameter, and according to* gender*, through a univariate model, we can observe that there is no interaction between tolerance and* gender*; that is, having good or bad tolerance does not depend on* gender*. Taking two parameters as a sample, we can observe that the increase in* heart rate* measured after the simulated resuscitation by* gender* is 14.98 bpm in males (95 percent CI 11.72 to 18.23) and 8.89 bpm in females (95 percent CI 6.03 to 11.76) with respect to the baseline measurement (*p* = 0.273).* Systolic blood pressure* values showed a mean decrease of 3.55 mmHg (95 percent CI -7.84 to 0.74) from the baseline in men and 4.95 mmHg (95 percent CI -8.10 to -1.80) in women (*p* = 0.902).  Table 5 shows the means and 95 percent confidence intervals corresponding to the changes observed in each variable, according to tolerance and* gender*, with its statistical significance.

In the application of the univariate model to each dependent variable, the interaction between tolerance and* gender* does not appear as statistically significant. This fact allows us to study* gender* separately. Having good or bad physiological tolerance does not depend on* gender*.

## 4. Discussion

Variation in the values of the vital signs did not present differences by* gender* that could indicate a parameter that varies significantly; on the contrary, a uniform pattern could be observed throughout the sample. Participants who tolerated the test well exhibited a decrease in* heart rate*,* systolic* and* diastolic blood pressure*, lactic acid, and temperature; on the contrary, they presented an increase in* perfusion index* and* hemoglobin* values. The subjects that did not tolerate the simulated resuscitation with the personal protection equipment for biological hazards showed deviations in the expected values in five or more parameters studied.

The physiological model explains certain alterations, such as the increase in* hemoglobin* and* perfusion index* values. The physiological response to intense exercise and the release of catecholamines leads to peripheral vasoconstriction [17] which involves a redistribution of blood flow, increasing the supply of red blood cells and, therefore, of saturated* hemoglobin* to central organs.

Despite the fact that once the participants had performed the simulation they removed their PPE following the protocol provided, and had a 10-minute rest,* heart rate *values after the test showed an average increase of 11.43 beats per minute compared to the baseline measurement. This fact is justified by the increase in physical activity and the release of catecholamines [18, 19].

During moderate or high intensity physical exercise, as resuscitation could be performed whilst wearing biohazard PPE,* blood pressure *increases to compensate for the increased demand for energy production. At the end of the physical exercise, generalized vasodilatation occurs and, consequently, redistribution of blood flow and thus decreasing* blood pressure*. About 5-6 minutes after physical exercise* blood pressure* decreases to baseline resting values, and it continues decreasing to lower values, keeping this decrease in the following 5-6 hours [20, 21].

If we combine thermal stress with the physiological overload caused by heat (due to the effort involved in resuscitation maneuvers), working with biohazard PPE significantly alters physiological thermoregulation mechanisms [22]. The health worker will begin to sweat, and consequently there will be evaporation inside the suit, causing the skin to cool. At the same time, blood flow to the skin increases (peripheral vasodilatation), to increase the release of heat to the outside, which is impossible in this case due to the waterproof characteristics of the protective equipment. If this situation is reached under a very intense or lasting physical effort, professionals who are wearing this equipment begin to feel uncomfortable and present diminished attention and response capacity [23].

Regarding the variation of lactic acid, it should be firstly mentioned that, in high intensity and short duration physical exercises (more the less trained the person is), the body does not have enough immediate available oxygen and must get energy by less efficient routes that generate more metabolic waste (glycolytic metabolism) [24]. Consequently, with high levels of lactic acid, the ability to generate energy decreases and so does muscle capacity, thus fatigue appearing early [25, 26].

After the analysis, no statistically significant association was found between a good or poor tolerance to the proposed experimentation protocol and genre. This means that it is easy to demonstrate that having to perform a job wearing biohazard PPE complicates procedures at a technical level and that the use of this equipment involves a high intensity physical effort and high intensity work performance that does not allow working for very long periods of time. The use of this equipment makes transpiration and thermoregulation very difficult and can raise workers' body temperature, who may also have vision and hearing problems and increased* heart rate* and* blood pressure* during the working time. This condition affects men and women equally.

## 5. Limitations

Like any research study, this one presents specific scientific objectives and has been carried out in a specific limited context, so the following limitations should be noted:

- The research is limited to the study of physiological and anthropometric parameters cited in the methodology, but the usefulness of other parameters such as cortisol, pH, or insulin levels is not discussed. These parameters were discarded from the study due to the complexity required to make the determinations.
- The study analyzes in detail the physiological behavior of the volunteers before a simulated resuscitation, but the study must be deepened to also evaluate the importance of the psychological component in the modulation of this physiological response.

## 6. Conclusions

A homogenous distribution of the physiological and anthropometric parameters was observed, and no significant differences were found by* gender*.

We can conclude that, depending on the parameters studied, there is a metabolic pattern of poor physiological tolerance after the use of level D PPE for the observed sample, which may lead us, in future studies, to derive a predictive rule that allows us to assess what professionals may have better adaptability and tolerance in their work in a biological incident situation, but not influenced by the worker's* gender*. Therefore, this so technical and specialized work can be performed by any properly trained professional.

## Figures and Tables

**Table 1:** Exclusion criteria to participate in the study.

Electrocardiogram showing any alteration	Body mass index greater than 40

Abnormal spirometry	Functional impotence

Baseline *heart rate* greater than 150 beats per minute	Baseline *heart rate* less than 35 beats per minute

*Systolic blood pressure* greater than 160 mmHg	*Diastolic blood pressure* greater than 95 mmHg

*Systolic blood pressure* less than 80 mmHg	*Tympanic temperature *greater than 38°C

Capillary glucose levels less than 65 mg/dL	Skin diseases during acute phase

Severe visual impairment	Severe hearing loss

**Table 2:** Distribution of *age* and body mass index of the participants by gender and physiological tolerance.

*Gender*	Tolerance	N	*Age* (years old)	BMI (kg/m^2^)
Male	Global	40	33.80 ± 11.80	25.95 ± 4.30
	Good T.	31	33.23 ± 11.88	26.12 ± 4.60
	Poor T.	9	35.78 ± 12.01	25.34 ± 3.21

Female	Global	56	29.54 ± 9.76	23.21 ± 3.62
	Good T.	43	30.81 ± 10.14	23.41 ± 3.63
	Poor T.	13	25.31 ± 7.15	22.58 ± 3.67

N: number; BMI: body mass index; T: tolerance.

**Table 3:** Distribution of vital signs before/after by physiological tolerance.

Good tolerance N= 74					
	Before		After		
	Mean	SD	Mean	SD	*p*
*HR* (bpm)	80.24	13.67	91.26	13.10	<0.001
*SBP* (mmHg)	131.31	14.28	124.28	15.05	<0.001
*DBP* (mmHg)	83.07	9.67	79.92	9.88	0.001
*TT* (°C)	36.69	0.47	37.19	0.39	<0.001
*Hb* (mg/dL)	13.54	1.50	14.24	1.50	<0.001
*PI* (%)	2.98	2.46	6.66	3.44	<0.001
*LA* (mmol/L)	2.62	1.70	3.65	2.46	0.001

Poor tolerance N = 22					
	Before		After		
	Mean	SD	Mean	SD	*p*

*HR* (bpm)	79.91	12.60	92.73	13.17	<0.001
*SBP* (mmHg)	123.64	12.77	128.23	15.04	0.127
*DBP* (mmHg)	74.27	7.02	78.32	6.86	0.002
*TT* (°C)	36.53	0.67	37.28	0.53	<0.001
*Hb* (mg/dL)	14.15	1.36	13.90	1.43	0.258
*PI* (%)	4.36	3.94	6.30	3.74	0.070
*LA* (mmol/L)	1.75	0.71	3.76	1.68	<0.001

N: number; TT: *tympanic temperature*; HR: *heart rate*; bpm: beats per minute; SBP: *systolic blood pressure*; DBP: *diastolic blood pressure*; Hb: *hemoglobin*; PI: *perfusion index*; LA: *lactic acid*; SD: standard deviation.

**Table 4:** Distribution of vital signs before/after by *gender*.

Male N= 40					
	Before		After		
	Mean	SD	Mean	SD	*p*
*HR* (bpm)	78.48	15.01	93.45	13.50	<0.001
*SBP* (mmHg)	137.73	12.70	134.18	13.12	0.112
*DBP* (mmHg)	85.08	9.97	82.65	9.52	0.049
*TT* (°C)	36.48	0.56	37.13	0.40	<0.001
*Hb* (mg/dL)	14.72	1.20	15.37	0.94	<0.001
*PI* (%)	4.37	3.23	7.80	3.53	<0.001
*LA* (mmol/L)	2.66	1.92	4.18	2.88	0.004

Female N= 56					
	Before		After		
	Mean	SD	Mean	SD	*p*

*HR* (bpm)	81.38	12.06	90.27	12.69	<0.001
*SBP* (mmHg)	123.71	12.39	118.77	12.99	0.003
*DBP* (mmHg)	78.18	8.73	77.34	8.49	0.450
*TT* (°C)	36.78	0.45	37.26	0.44	<0.001
*Hb* (mg/dL)	12.94	1.20	13.30	1.16	0.022
*PI* (%)	2.53	2.38	5.70	3.23	<0.001
*LA* (mmol/L)	2.26	1.25	3.31	1.71	<0.001

N: number; *TT*: *tympanic temperature*; *HR*: *heart rate*; bpm: beats per minute; *SBP*: *systolic blood pressure*; *DBP*: *diastolic blood pressure*; *Hb*: *hemoglobin*; *PI*: *perfusion index*; *LA*: *lactic acid*; SD: standard deviation.

**Table 5:** Distribution of the means of the differences of the measured values and their confidence intervals according to tolerance and *gender*, as well as their statistical significance and *p* values of the created models.

	Gender	Mean	SD	95% IC	*p*
*HR* (bpm)	Male	14.98	10.49	11.72; 18.23	0.273
	Female	8.89	10.94	6.03; 11.76	

*SBP* (mmHg)	Male	-3.55	13.84	-7.84; 0.74	0.902
	Female	-4.95	12.02	-8.10; -1.80	

*DBP* (mmHg)	Male	-2.43	7.57	-4.77; -0.08	0.798
	Female	-0.84	8.26	-3.00; 1.32	

*TT* (°C)	Male	0.66	0.48	0.51; 0.80	0.811
	Female	0.48	0.42	0.37; 0.59	

*Hb* (mg/dL)	Male	0.66	0.96	0.36; 0.95	0.279
	Female	0.36	1.14	0.06; 0.66	

*PI* (%)	Male	3.43	3.97	2.20; 4.66	0.608
	Female	3.18	3.62	2.23; 4.12	

*LA* (mmol/L)	Male	1.53	3.18	0.54; 2.51	0.122
	Female	1.05	1.85	0.57; 1.54	

N: number; *TT*: *tympanic temperature*; *HR*: *heart rate*; bpm: beats per minute; *SBP*: *systolic blood pressure*; *DBP*: *diastolic blood pressure*; *Hb*: *hemoglobin*; *PI*: *perfusion index*; *LA*: *lactic acid*; SD: standard deviation.

## Data Availability

The DA statement for 5890535 should be "The data that has been used is confidential, the participants in the study are active members of the emergency services and data about their medical history can not be disseminated.
